# COVID-19 and mutations a threat level assessment

**DOI:** 10.3126/nje.v11i1.35659

**Published:** 2021-03-31

**Authors:** Jared Robinson, Indrajit Banerjee, Alexandra Leclézio, Brijesh Sathian

**Affiliations:** 1-3 Sir Seewoosagur Ramgoolam Medical College, Belle Rive, Mauritius; 4 Geriatric and long term care Department, Rumailah Hospital, Hamad Medical Corporation, Doha, Qatar

**Keywords:** COVID-19 Virus variants, SARS-CoV-2 B.1.351, SARS-CoV-2 B.1.1.7 variant, SARS-CoV-2 variants

## Abstract

A mutation is defined as an alteration in the DNA or RNA sequences of a genome which may consequently confer a new phenotypic and or genotypic advantage both increasing the virulence as well as the survival of a virus or pathogen. At this current point in time there are 4 known major variants of the original SARS-CoV-2 virus, namely the English variant (B.1.1.7), the South African variant (B.1.351), Brazilian variants (VOC202101/02 (P.1) and VUI202101/01) and a variant similar to that of the South African variant found in North America (B.1.526), all of which have varying levels of resistance and infectivity. It is evident that the SARS-CoV-2 variants pose an international health risk, the mutations of E484K and N501Y are the two most implicated mutations. E484K being the most concerning as it aids in immune evasion and drastically causes the efficacy of the current vaccines to be reduced by large margins. The most worrisome variant is the South African or B.1.351 which harbors the above mutations. It is of the upmost importance that targeted vaccines are synthesized to ensure that immunized individuals have effective protection against these variants. Until these specific targeted vaccines are synthesized the current vaccines offer little long-term protection, however do confer a level of immunity to stop severe infections. It is thus advised that current vaccination programs should continue in earnest as a degree of protection is conferred.

## Background

A mutation is defined as an alteration in the DNA or RNA sequences of a genome which may consequently confer a new phenotypic and or genotypic advantage both increasing the virulence as well as the survival of a virus or pathogen. Mutations thus pose a threat to the pharmacotherapy of various conditions and play a poignant role in drug resistance for example MDR-TB and XDR-TB. It is now evident due to the discovery of multiple mutations in the SARS-CoV-2 virus that the treatment, prevention and international relations between countries harboring various strains may have to be reviewed until a true risk assessment of the mutations can be accurately quantified [1,2]. What risks do these new mutations pose? Will the current vaccination scheme be effective?

### SARS-CoV-2 virology

Coronaviruses were donned so due to the crown-like glycoprotein spikes that are visible on their surface under electron microscopy. For the most part these coronaviruses cause relatively benign infections, for example HCoV-OC43, HCoV-NL63, HCoV-229E and HCoV-HKU1 are known to produce a flulike syndrome. The Severe acute respiratory syndrome coronavirus (SARS) and Middle Eastern respiratory syndrome coronavirus (MERS) coronaviruses however cause more severe and life-threatening infections as well as manifestations. The phylogenetic analysis divides Coronaviruses into four genera, namely Alpha-CoV, Beta -CoV, Gamma-CoV and Delta -CoV. The Beta- CoV genus is divided into 4 lineages, now being referred to as Groups A, B, C and D. SARS-CoV-2 has been sequenced and subsequently found to be part of the Beta coronaviruses and has been placed in lineage B [3-7].

### SARS-CoV-2 variants

At this current point in time there are 4 known major variants of the original SARS-CoV-2 virus, namely the English variant, the South African variant, the Brazilian variant and a variant similar to that of the South African variant found in North America, all of which have varying levels of resistance and infectivity.

### Brazilian variants [VOC202101/02 (P.1) and VUI202101/01]

Brazil has 2 variants namely: VOC202101/02 (P.1) and VUI202101/01. Of the two variants the VUI202101/01 variant has less mutations and poses a significantly reduced threat to public health as opposed to the P.1 variant. The VUI202101/01 variant was discovered in the United Kingdom in the first half of January 2021.

The P.1, VOC202101/02 variant was first discovered in Japan in individuals who had arrived from Brazil. This strain had mutations and or variations present in more than 16 amino acids. Three of which being deletions. There are a host of phenotypically important mutations namely some being mutations of E484K and N501Y both of which involve the glycoprotein spikes of the virus.

The N501Y mutation is shown to confer an enhanced virulence in animal study models using mice. It must be noted that this mutation is also present in the English variant. The E484K mutation is found in the South African variant and confers greater resistance to antibodies, and thus poses a greater threat. It is thus evident that the P.1 strain has the mutations of both E484K and N501Y making it more virulent as well as more resistant to antibodies. It is thus of a far greater concern to international public health as it harbours mutations which increase both its ability to be trans-missed as well as its ability to withstand a hosts immune response [8,9].

### The South African Variant [B.1.351]

B.1.351 or more commonly known as the South African variant was discovered at a similar time to that of the English variant. This variant however is cause for a much greater concern. It has spread rapidly and now has been identified in over 20 countries. This variant has outnumbered the conventional infections in South Africa due to its greater viral load, shedding and higher resistance. A study of the genome of this B.1.351 variant has revealed very similar mutations to those present in the P.1 Brazilian strain, namely mutations of E484K and N501Y glycoproteins are present. These mutations thus conferring an increased virulence and greater resistance to antibodies. The mutations that are present in both the P.1 and B.1.351 variants are thus of the greatest international concern and risk. The South African strain is of particular international concern as it has spread to over 20 countries and has dominated local infections, thus acting as an international watchdog for future mutations and their enhanced ability to spread rapidly. A cause for further concern is the diminished efficacy shown by vaccines such as the Oxford AstraZeneca against this variant, which has caused a complete shift in the South African vaccination strategy [10-12].

### The English strain [B.1.1.7]

The English variant contains a host of mutations but most poignant is the mutation of N501Y which confers a higher transmissibility of the virus, via increasing viral shedding and load. The N501Y causes an asparagine □ tyrosine AA substitution at position 501 present in the S gene. This same mutation has been identified in both the Brazilian P.1 as well as the South African strain. It must be noted that the new strain has shown an increase in symptoms such as dysphagia, myalgia and fatigue [13,14].

### The [B.1.526] variant discovered in New York

The B.1.526 variant was discovered whilst PCR testing and subsequent genomic analysis in a pool of COVID patients in New York. It was found that individuals infected with the B.1.526 variant were generally older in age and often were more likely to develop more serious complications requiring hospitalization. The B.1.526 variant has a mutation of the E484K conferring a greater resistance to antibodies and thus poses a great concern. It was also discovered that the B.1.526 variant has scattered clusters in the Northeastern aspect of the United States. It is believed that it’s set of mutations confer the virus’s unique phenotypic nature which is similar to that of the South African and P.1 variant and is thus cause for a major international health concern [15,16].

### Variants and vaccine efficacy

With the world well underway with various vaccinations programs, the emergence of these mutants raises great concern for vaccine efficacy and the level of protection that the active immunization confers.

It is now evident that the Oxford AstraZeneca vaccine has been said to have limited efficacy against the South African strain and has thus caused the South African government to stop its immunization campaigns. The Johnson and Johnson vaccine is now being used to immunize frontline workers in South Africa. An FDA report states an efficacy of 57% in South Africa and a higher 66% efficacy in South America. The efficacy is calculated in terms of preventing moderate to severe infections [17,18].

The Novavax vaccine has shown promising trial data with a 95.6% efficacy against the original strain and a 60% efficacy against the South African variant and an even higher 85.6% efficacy against the English variant [19].

In a study published by the New England Journal of Medicine it has been shown that the Pfizer and BioNTech vaccine had a two third reduction in its ability to neutralize antigens of the B.1.351 variant. This proving that all of the available vaccines on the market have a reduced efficacy against the new variants. It believed that the immunity conferred against the B.1.351 variant is not long lasting and functions more so as a therapy to decrease spread as opposed to providing immunity against the virus. It must be noted however that all 3 major companies are working on modifications of the vaccines which are specifically targeted towards the new variants. It has however been shown that immunization with any vaccine does reduce the risk of severe life-threatening infections and complications [20,21].

### Threat level assessment

At this current stage the South African variant B.1.351 poses the greatest risk to international health as it is the most widespread, most resistant to current vaccinations and harbours both the E484K and N501Y mutations. It has also been shown to have a poor response to treatment and the vaccine efficacy is low, with vaccines now being used to better curb the spread of the virus as opposed to conferring actual long lasting host immunity [17,22].

The P.1 and the B.1.526 variant discovered in New York also harbour the worrisome E484K mutation and thus also pose a great risk, however they are less quantified whence compared to the South African strain. It is poignant however to ensure that targeted vaccines are synthesized to combat the strains harbouring the E484K mutation as it confers the ability to escape the immune response and thus cause a more severe and life-threatening infection [23,24].

A large benefactor which increases the risk level of the P.1, B.1.526 and B.1.351 variants is the fact that they effect the elderly more readily and thus this increases the likelihood of these variants causing catastrophic complications and death in an already compromised host who already most likely has underlying comorbidities [15,16].

The English or B.1.1.7 harbours the N501Y mutation which confers a higher transmissibility of the virus and greater shedding of virions. It is of a less quantitated risk and current vaccination programs are yielding effective results and thus this reduces the risk level of this variation by a great margin [7,25].

### Expert opinion

The emergence of new variants in an already novel virus brings about a host of new challenges and complications. The foremost complication being the question in matter as to whether current immunization strategies will prove fruitful and effective towards these new variants. It has now been established that the P.1, B.1.526 and B.1.351 variants pose the greatest risk to international health as they harbor the infamous E484K mutation, thereby enhancing the capability of the virus to escape the host immune response. All of the current vaccines available on the international market have shown a large decrease in efficacy when used against the B.1.351 variant, and now are believed to only confer short term protection and thus more so act as a therapy to curb the spread of the virus. It must be noted however that any vaccination at this point in time will be beneficial in nature as the immunization reduces the likelihood of severe and life-threatening infections.

## Conclusion

It is evident that the SARS-CoV-2 variants pose an international health risk, the mutations of E484K and N501Y are the two most implicated mutations. E484K being the most concerning as it aids in immune evasion and drastically causes the efficacy of the current vaccines to be reduced by large margins. The most worrisome variant is the South African or B.1.351 which harbors the above mutations. It is of the upmost importance that targeted vaccines are synthesized to ensure that immunized individuals have effective protection against these variants. Until these specific targeted vaccines are synthesized the current vaccines offer little long-term protection, however do confer a level of immunity to stop severe infections. It is thus advised that current vaccination programs should continue in earnest as a degree of protection is conferred.
